# An Overview on the Clinical Development of Tau-Based Therapeutics

**DOI:** 10.3390/ijms19041160

**Published:** 2018-04-11

**Authors:** Miguel Medina

**Affiliations:** Network Center for Biomedical Research in Neurodegenerative Diseases (CIBERNED), Queen Sofia Foundation Alzheimer Center, CIEN Foundation, Carlos III Institute of Health, 28031 Madrid, Spain; mmedina@ciberned.es; Tel.: +34-913-852-200

**Keywords:** aggregation, Alzheimer, dementia, drug development, immunotherapy, neurodegeneration, tau, tauopathies, therapy

## Abstract

Tauopathies such as Alzheimer’s disease (AD), frontotemporal lobar degeneration, or progressive supranuclear palsy constitute a group of brain disorders defined by neurodegeneration and the presence of tau aggregates in the affected brains regions. Tau is a microtubule-associated protein that accumulates in the cytosol under pathological conditions, steering the formation of aggregates or inclusions thought to be involved in the degeneration and neuronal death associated with these diseases. Despite a substantial and unmet medical need for novel, more effective disease-modifying therapies for the treatment of AD and tauopathies, the last couple of decades have seen numerous drug development undertakings primarily focused on β-amyloid, with disappointing results to date. On the other hand, tau-focused approaches have not received much attention until recently, notwithstanding that the presence of extensive tau pathology is fundamental for the disease and tau pathology shows a better correlation with impaired cognitive function than with amyloid pathology in AD patients. The last few years have brought us advances in our comprehension of tau biological functions beyond its well-established role as a microtubule-associated protein, unveiling novel physiological tau functions that may also be involved in pathogenesis and thus provide novel targets for therapeutic intervention. This review describes several emerging, encouraging therapeutic approaches aimed at tackling the underlying causes of tau pathology in AD and other tauopathies that have recently reached the clinical development stage.

## 1. Introduction

First isolated as a heat-stable protein essential for tubulin assembly into microtubules (MT) [1], tau protein was later clearly established as an axonal microtubule-associated protein (MAP) in neurons that, under physiological conditions, regulates microtubule assembly, dynamic behavior, and spatial organization [2] as well as axonal transport of organelles [3]. It had been previously isolated from the paired helical filaments of neurofibrillary tangles present in the brain of Alzheimer’s disease (AD) patients [4] that are made up of abnormally hyperphosphorylated tau [5]. A number of post-translational modifications, including phosphorylation, acetylation, or truncation, have been shown to play important roles in tau function and dysfunction [6]. Moreover, studies in recent years have increasingly unveiled novel physiological tau functions apart from its well-established functions in MT stabilization and axonal transport, which may also play a role in pathogenesis [7].

In the central nervous system of mammals, tau protein is composed of six different isoforms produced by alternative splicing mechanisms from a single *MAPT* gene located at chromosome 17q21 [8]. Three of these isotypes contain three copies of the imperfect 31 amino acid repeats that constitute the microtubule-binding domain (tau 3R), whereas the other three isotypes contain four repeats (tau 4R) [9].

Intracellular tau aggregates are a common finding in a number of neurodegenerative disorders, collectively referred to as tauopathies, generally associated with synaptic loss and neuronal death, including AD, frontotemporal lobar degeneration (FTLD), progressive supranuclear palsy (PSP), corticobasal degeneration (CBD), and Pick’s disease [10]. The identification of some families bearing highly penetrant, dominant mutations within the *MAPT* gene causing frontotemporal dementia [11] demonstrated that tau dysfunction is sufficient to cause neurodegeneration and dementia. Some mutations within the *MAPT* gene disrupt the interaction between tau and microtubules, while splicing mutations of *MAPT* exon 10 lead to changes in the ratio between 3R and 4R isoforms [10], emphasizing the role of alternative splicing in tau dysregulation.

Despite the fact that neurofibrillary tau pathology correlates much more closely with clinical symptoms of dementia than amyloid pathology, drug discovery and development efforts for AD in the last two decades have primarily focused on targets related to β-amyloid, so far with highly disappointing results. In contrast, tau-based strategies have received little attention until recently, despite that the presence of extensive tau pathology is central to the disease. However, a number of different strategies targeting several aspects of the tau-dependent pathogenesis are being actively pursued at present, some already in advanced clinical development [12,13,14], thus broadening our range of potentially useful therapeutic tools to treat AD and other tauopathies.

Here we provide an overview of the most recent developments regarding the various points of therapeutic intervention with disease-modifying potential that are being pursued in clinical development for AD and other tauopathies.

## 2. Modulating Tau Phosphorylation

The robust correlation existing between tau phosphorylation and pathology has provided the groundwork for the search for tau protein kinase inhibitors as potentially useful therapeutic agents. Considering that in vivo tau hyperphosphorylation is most likely the result of multiple protein kinase activities [15,16,17], whether the more effective target for reducing pathological tau phosphorylation is a specific kinase or distinct groups of protein kinases has remained an open question [18]. Several protein kinases, including GSK-3β, MARK, and CDK5, have all been proposed as potential therapeutic targets [19]. Notwithstanding the challenges faced by such approaches, especially concerning the issues of toxicity and specificity, developing protein kinase inhibitors has continued to be an area of intense preclinical efforts in recent years. The most advanced protein kinase inhibition strategy in the clinic so far has been aimed at GSK-3β [20,21].

It has been generally presumed that a meaningful proportion of the therapeutic effects observed with the mood stabilizing drug lithium is due to the inhibition of GSK-3, although the compound has other effects that cannot be disregarded, in particular on the inositol pathway. Thus, a handful of observational pilot studies have made an effort to address the clinical effect of lithium in patients with either AD or mild cognitive impairment, with inconsistent results partially attributable to low number of subjects recruited, low compliance, and narrow therapeutic window [22,23].

Tideglusib (NP031112, NP-12) is an orally available GSK-3β inhibitor [24] that in preclinical studies reduced tau phosphorylation, neuronal loss, and gliosis, and reversed a spatial memory deficit in transgenic mice [25]. Thirty patients with mild to moderate AD were treated for 20 weeks with tideglusib in a placebo-controlled, escalating-dose phase IIa clinical trial (NCT00948259), showing positive trends on the MMSE and ADAS-Cog tests [26]. A subsequent 6-month, phase IIb trial in 308 patients with mild to moderate AD was conducted in 55 centers across several European countries (NCT01350362). Tideglusib proved safe in the trial but missed its primary endpoint and some of the secondary endpoints, thus showing no significant clinical benefit [27]. The compound received U.S. food and Drug Administration (FDA) and European Medicines Agency (EMA) orphan drug status and was also evaluated in a 1-year study in 146 patients with PSP (NCT01049399). Again, this study did not meet the primary endpoint [28], although signs of decreased brain atrophy were reported in a subgroup analysis [29].

Another protein kinase that has received increasing attention as a potential therapeutic target is the tyrosine kinase Fyn, which phosphorylates tau in the N-terminal domain in dendrites and also has a role in the amyloid signaling pathway [30]. The repurposed small molecule saracatinib (AZD0530) is a Fyn inhibitor shown to reduce memory deficits in transgenic mice and reported to be safe and well tolerated in a phase I clinical trial (NCT01864655) [31]. A phase IIa multicenter study in 159 mild AD patients treated with saracatinib is still ongoing (NCT02167256).

On the other side of the phosphorylation coin are the phosphatases. Hence, activation of protein phosphatases has also been suggested as an additional strategy to reduce tau phosphorylation, especially in the instance of protein phosphatase 2A (PP2A), the main brain phosphatase involved in tau phosphorylation [32]. Activation of PP2A is particularly challenging though, as it has broad substrate specificity and several regulatory subunits, which makes it difficult to identify the right pool of PP2A to be targeted to the right extent in the right place and time [33].

## 3. Targeting Other Tau Post-Translational Modifications

Apart from phosphorylation, tau is also extensively post-translationally modified by lysine acetylation, which leads to impaired tau function and promotes pathological aggregation, which in turn has prompted proposing the use of tau acetylation inhibitors as a potential therapeutic strategy for AD and other tauopathies [6]. Thus, salsalate—a nonsteroidal anti-inflammatory drug (NSAID) approved by the FDA to treat arthritis and other conditions that cause swelling—has been shown to inhibit acetyltransferase p300-induced tau acetylation and improve tau-mediated behavioral impairments and neurodegeneration in an frontotemporal dementia (FTD) mouse model [34]. This compound is about to complete a 6-month multicenter, open label, pilot futility phase I clinical trial to assess the safety, tolerability, pharmacodynamics, and preliminary efficacy of orally administered salsalate in 10 patients with PSP (NCT02422485). More recently, a phase 1b clinical trial has been launched with the aim of evaluating its safety and tolerability in patients with prodromal to mild AD (NCT03277573). Forty participants will be randomized to receive either a placebo or salsalate twice daily for 1 year. Outcomes include changes in cerebrospìnal fluid (CSF) biomarkers, brain MRI, and cognitive function. The study is expected to be completed by the end of 2018.

Another tau post-translational mod ification is glycosylation since tau has been shown to be *O*-GlcNAc-modified in human brain. In particular, the carbohydrate *N*-acetylglucosamine (GlcNAc) can form β-glycosidic *O*-links with serine and threonine residues on tau. This reversible process is regulated by two main enzymes: *O*-GlcNAc transferase (OGT) inserts *O*-GlcNAc, while *O*-GlcNAcase (OGA) eliminates this modification from proteins. Since in certain cases GlcNAc may affect phosphorylation and aggregation of tau protein [35], it has been hypothesized that increasing tau *O*-GlcNAc could be a sensible strategy to hamper neurodegeneration brought by pathological tau. In fact, OGA modulators have been developed as a disease-modifying strategy for tauopathies.

As a matter of fact, the selective and potent small-molecule inhibitor of the OGA enzyme known as MK-8719 has recently entered into early clinical trials for the treatment for PSP. Clinical development of this compound was initiated with a single ascending dose phase I study in 16 healthy volunteers, with the objective of evaluating the compound’s safety, tolerability, and plasma pharmacokinetics. The study revealed promising human pharmacokinetics and pharmacodynamic effects of OGA inhibition in blood by measuring the *O*-protein in peripheral blood mononuclear cells (PBMC). Moreover, brain OGA target engagement was assessed in a second phase I study, taking advantage of the suitability of the labeled compound (^18^F-MK-8553) as a positron emission tomography (PET) tracer [36,37]. The results obtained support further clinical development and evaluation of safety and efficacy in patients with PSP.

ASN120290 (previously ASN-561) is a brain-permeable small-molecule *O*-GlcNAcase enzyme inhibitor that has just initiated a phase I clinical trial in healthy volunteers, following regulatory approval, as presented at the 13th International Conference on Alzheimer’s and Parkinson’s Diseases recently held in Vienna. The randomized, double-blind, placebo-controlled phase I study was designed to assess the safety, tolerability, food effect, pharmacokinetics, and pharmacodynamics of single and multiple doses of the compound. The assessment of a blood-based biomarker in the trial will support the optimal dose selection of ASN120290 for future studies. Furthermore, a phase II proof-of-concept study for the treatment of the orphan tauopathy PSP is being planned after the successful completion of the phase I trial.

Genome-wide association studies have revealed common variants of the gene *EIF2AK3*, which encodes the double-stranded RNA-activated protein kinase-like endoplasmic reticulum kinase (PERK) that increases the risk for PSP [38]. Neuropathological evidence has emerged implying the involvement of anomalous PERK signaling in the pathogenesis of tauopathies. PERK constitutes an integral part of the unfolded protein response (UPR). Tau accumulation in cells could activate PERK, which in turn activates GSK-3β, but the precise molecular mechanisms of PERK function in tau pathophysiology are not fully understood yet [39]. Nevertheless, studies on animal models strongly suggest that PERK activation may be a feasible approach for treating PSP as well as other tauopathies. Actually, the small molecule PERK activator CCT020312 is about to initiate phase I clinical trials.

Although not much information is available regarding the specific mechanism of action, AZP2006 is an orally available drug candidate that has been given orphan drug status by the EMA for the treatment of PSP. According to the EMA opinion on orphan designation for AZP2006, the drug appears to work by blocking tau phosphorylation and preventing it from folding incorrectly. In addition, it seems to stimulate macroautophagy, thus helping to clear and eliminate misfolded proteins. The first phase I clinical trials on healthy volunteers (males aged between 18 and 55) was initiated in 2015. These randomized, placebo-controlled studies allowed evaluating the safety, tolerability, and pharmacokinetics of several doses of AZP2006 during a short 10-day treatment. AZP2006 has been well tolerated and, in 2016, additional studies were launched on healthy volunteers (males and females aged over 65) using a longer 28-day treatment period. The compound is finishing phase I studies for PSP and AD in France, although no detailed results are available yet. A phase II study on PSP is planned for 2018.

Nilotinib is a c-Abl tyrosine kinase inhibitor approved for use in patients with leukemia [40]. The rationale for using nilotinib is based on research regarding clearance of tau and other proteins accumulated in the brain of patients affected by neurodegenerative diseases. Although the precise molecular mechanism is not known in full detail, nilotinib appears to penetrate the blood–brain barrier and induce autophagy inside neurons to clear not only tau, but also amyloid-β and other toxic proteins. Promising results in a small study undertaken in 2015 in a few Parkinson’s disease patients (NCT02281474) provided the basis for a phase II randomized, double-blind, placebo-controlled trial to assess the impact of nilotinib on safety, biomarkers, and clinical outcomes in 42 patients diagnosed with mild to moderate AD (NCT02947893). The study’s primary objectives are to test the drug’s safety and tolerability (orally administered once a day) after 12 months and to measure whether nilotinib reduces inflammation and the presence of β-amyloid and tau in spinal fluid. Cognitive and functional ability tests will also be performed. Estimated completion date is set to be by the end of 2019.

## 4. Microtubule Stabilizers

The idea that tau detachment from microtubules leads to a loss of its normal MT-stabilizing function and may eventually result in axonal transport defects and synaptic dysfunction has led to proposing the use of MT-stabilizing molecules as therapeutic agents. Nevertheless, whether MT destabilization is an early and/or relevant event directly involved in tau toxicity in AD or other tauopathies is still under debate [14]. Actually, whereas the effect of tau in promoting MT assembly is unquestionable, a direct involvement in impairing axonal transport under physiological conditions in vivo is a controversial issue in light of some gain-of-function and loss-of-function studies in mice [41]. Interestingly, it has been reported that Aβ oligomers are able to destabilize MT and disrupt fast axonal transport via calcineurin activation in tau-deficient mice, which has led to the idea that MT destabilization might be a general downstream event during neurodegeneration [42].

Among those MT-stabilizing molecules, some antimitotic drugs such as paclitaxel or epothilone D have been tested in tau-overexpressing mouse models. Epothilone D (BMS-241027) is a taxol-derived small-molecule microtubule stabilizer that readily crosses the blood–brain barrier and has been described as restoring the spatial memory deficits by reducing tau pathology and hippocampal neuronal loss in transgenic animals [43]. The compound was tested in 2013 in a phase I multicenter, randomized, double-blind, placebo-controlled clinical study (NCT01492374) to assess its safety, tolerability, and pharmacodynamics in 40 patients with mild AD, but no results have been published and drug development efforts for AD have been halted.

More recently, a novel small molecule derived from taxol, termed as abeotaxane or TPI-287, was tested in a phase I trial to address safety and tolerability in 44 patients with CBD or PSP (NCT01966666) and 33 AD patients (NCT02133846). Abeotaxane was administered by intravenous infusion for 9 weeks (once every 3 weeks), with an option for open-label extension up to 3 months. As presented in the last Clinical Trials on Alzheimer’s Disease (CTAD) meeting in Barcelona in December 2017, the treatment was not well tolerated by AD patients and led to some undesired side effects in the CBD/PSP cohort. Exploratory cognitive endpoints did not show any significant improvement.

The microtubule-stabilizing agent that has reached the most advanced clinical development status is davunetide (also known as NAP or AL-108)—an eight-amino acid peptide arisen from the activity-dependent neuroprotective protein (ADNP)—that has produced promising effects on memory and behavior in tau transgenic mice [44]. Davunetide safety and tolerability profile was established after intranasal or intravenous administrations to mild cognitive impairment (MCI) subjects [45], but a later phase II/III clinical trial in over 300 PSP patients (NCT01110720) missed all endpoints [46]. A small pilot study on 12 subjects with various predicted tauopathies including PSP, FTDP-17, and CBD appears to be still active (NCT01056965).

## 5. Tau Aggregation Inhibitors

Tau aggregation and the various tau species formed (monomers, oligomers, prefilaments, granules, fibrils, insoluble aggregates) along the aggregation process have also been a focus of interest for potential therapeutic intervention. Hence, the use of in vitro and/or cell-based screening assays have permitted the identification of several small-molecule chemical families capable of inhibiting tau aggregation (reviewed in [47]). Although these compounds have shown efficacy through various in vitro assays, evidence of efficacy for inhibiting tau aggregation in vivo and significant cognitive improvement is for the most part lacking. 

Nevertheless, a proprietary formulation of the non-neuroleptic phenothiazine methylene blue (methylthioninium chloride)—a compound that has been used for quite some time for the treatment of malaria [48] and is FDA-approved for a number of conditions [49]—has moved up the clinical development ladder in recent years. The compound (also termed as Rember or TRx0014) readily crosses the blood–brain barrier and prevents tau aggregation in vitro as well as in cell and animal models [50]. A single-center, 24-week, dose-ranging monotherapy trial on 321 mild to moderate AD patients not taking acetylcholinesterase nor memantine (NCT00515333) was reported to have shown signs of benefit in moderate, but not mild, AD [51]. Although, the methodology, blinding status, and claims of efficacy have been highly criticized.

Since then, a second-generation replacement formulation of a stable reduced form of the methylthioninium moiety (leuco-methylthioninium bis-hydromethanesulfonate, LMTM; also known as LMT-X or TRx0237) has reached phase III clinical trials for the treatment of AD. A randomized, controlled, double-blind, parallel-group, multicenter trial aimed at determining safety and efficacy in 833 mild to moderate AD patients after 15 months of twice daily oral treatment (NCT01689246) showed no signs of efficacy [52]. More recently, a second 18-month phase III trial in 700 subjects with mild AD that were treated orally twice daily (NCT01689233) showed no effect on the primary endpoints [53]. Authors’ claims of efficacy after a post-hoc subgroup secondary analysis have been highly controversial, mainly concerning the methodology used and the interpretation of the results. Finally, a multinational, randomized, placebo-controlled, double-blind, parallel-group clinical trial intended to demonstrate safety and efficacy after 12-month oral treatment has been very recently completed in 220 patients with behavioral variant frontotemporal dementia (bvFTD) (NCT01626378), although results remain to be published [54].

It is worth mentioning that methylene blue (and davunetide for that matter) are known to have pleiotropic effects, so some authors have argued that sensu stricto they should not be considered primarily as tau-based therapies [55].

## 6. Anti-Tau Immunotherapy

Despite disappointing results from quite a few advanced clinical trials testing anti-amyloid immunotherapy for the treatment of AD [56], immunotherapies for diverse neurodegenerative disorders have become very actively sought after as a promising approach for the clearance of pathological proteins in these disorders. More recently, various anti-tau immunotherapy strategies have also been successfully tested in animal models, suggesting that this could be a feasible option for clearing toxic protein species in tauopathies, AD, and related disorders [57].

Anti-tau active immunotherapy strategies are based on eliciting specific antibody responses capable of clearing tau pathological species that could eventually result in neuronal function improvement. Hence, choosing the right epitope is crucial for a successful immunotherapy approach. Since hyperphosphorylation has been generally regarded as a candidate for inducing tau aggregation and neurofibrillary pathology, many phospho-epitopes have been tested in animal models with positive results. Nevertheless, tau also undergoes various modifications other than phosphorylation during the process from an intrinsically disordered soluble protein into insoluble aggregates and deposits. As discussed above, this cascade of events leading to neurofibrillary pathology might involve post-translational modifications such as phosphorylation, truncation, glycosylation, and ubiquitination [58].

Some of the tau immunotherapy clinical trials described below target various tau domains rather than any specific phospho-epitopes. Hence, it is worth mentioning that normal tau molecules can be sequestered by hyperphosphorylated tau into aggregates [59], eventually leading to pathological conformations in a prion-like manner [60,61].

Evidence has emerged in recent years in various animal models strongly suggesting that targeting abnormally phosphorylated tau epitopes (or specific pathologically relevant conformational epitopes) is a feasible approach to bring about antibody responses that in turn are able to promote tau clearance [21]. That has set the basis for a number of active and passive immunotherapy programs to reach clinical trials in humans for the treatment of AD and other tauopathies.

AADvac-1 is an active immunotherapy containing a synthetic tau peptide spanning residues 294–305 derived from a naturally occurring truncated and misfolded tau protein coupled to keyhole limpet hemocyanine (KLH) and aluminum hydroxide as adjuvant. It has recently completed phase I clinical trial (NCT01850238). In this first-in-man, randomized, double-blind, placebo-controlled study, 30 mild to moderate AD subjects aged 50–85 years received AADvac-1 subcutaneous injections for 12 weeks, showing a favorable safety profile and high anti-tau antibody titers [62]. An ongoing follow-up study continues to observe patients who have completed the phase 1 trial of AADvac-1 and receive additional immunization doses for another 18 months (NCT02031198). Recruitment of 185 patients with mild to moderate AD in a 24-month, randomized, placebo-controlled, parallel-group, double-blinded, multicenter, phase II safety and efficacy clinical trial for AADvac-1 started in 2016 (NCT02579252). The study design includes safety as the primary outcome, and cognitive and clinical assessments as well as a measure of immunogenicity as secondary outcomes. Other exploratory outcomes comprise FDG PET, MRI volumetry, and CSF biomarkers. The study is programed to run until 2019. More recently, a 24-month pilot study to evaluate the safety and immunogenicity of AADvac-1 in 30 subjects with the non-fluent variant of primary progressive aphasia (naPPA) has been launched (NCT03174886). Most cases of naPPA are associated with pathological changes found in FTD involving tau protein [63].

ACI-35 is a liposomal-based palmitoylated phospho-tau peptide—a synthetic peptide spanning the human protein tau sequence 393–408, phosphorylated at S396 and S404 [64]—that has been shown to reduce phospho-tau aggregates and total pathological tau and improve some cognitive parameters in animal models. It also elicited very specific tau antibody and tau T-cell independent immune response. A phase Ib multicenter, double-blind, randomized, placebo-controlled study to address the safety, tolerability, and immunogenicity of ACI-35 in subjects diagnosed with mild to moderate AD is currently ongoing (ISRCTN13033912).

Intravenous immunoglobulin (IVIg) is a human plasma-derived product consisting of polyclonal serum IgG pooled from thousands of blood donors that has been employed for quite some time and proven effective as anti-inflammatory and immunomodulatory therapy for various neurological diseases [65]. A phase III, randomized, double-blind, placebo-controlled trial (NCT00818662) in 390 participants with mild to moderate AD did not improve cognition or function after IVIg infusions performed every 2 weeks for 18 months [66]. Two more phase II (NCT01300728) and III clinical trials (NCT01561053) for the treatment of AD are still ongoing. Interestingly, tau-specific antibodies were found to be present in the IVIg product Flebogamma^®^ and recognized a recombinant tau fragment spanning residues 155–421 as well tau aggregates from AD brains [67].

To complement the active immunotherapy approaches just described, passive immunotherapy strategies are also being pursued using various anti-tau antibodies. This approach has been shown to be able to improve behavioral and cognitive impairments in mouse models [68]. Up to three tau-based passive immunotherapy programs have moved into clinical trials in the last few years, mostly for the treatment of AD and PSP, as described below.

BMS-986168 (BIIB092, IPN007) is a humanized IgG4 monoclonal antibody against extracellular, N-terminal tau fragments initially isolated from pluripotent stem cells obtained from a familial AD patient [69,70]. A first-in-man randomized, double-blind, placebo-controlled, single ascending dose-escalation clinical trial was completed in 2016 to investigate safety, tolerability, pharmacokinetics, pharmacodynamics, and immunogenicity in 65 healthy volunteers after intravenous infusion of BMS-986168 (NCT02294851). A second phase I multiple ascending dose study to assess the safety and tolerability in 48 patients with PSP has been recently completed (NCT02460094). This study also assessed the pharmacodynamics of BMS-986168 on CSF extracellular tau concentrations. Although the results of these two studies have not been published yet, an extension of the latter trial is currently ongoing with the same participants as a long-term treatment, open-label study that will run until 2020 (NCT02658916). A new randomized, double-blind, placebo-controlled, parallel-group phase II clinical trial to assess the efficacy and safety of BMS-986168 in 396 participants with PSP began enrolling during 2017 (NCT03068468). Participants will receive intravenous infusion of BMS-986168 once every 4 weeks for 208 weeks and the study is slated to run until 2020.

ABBV-8E12 (C2N-8E12) is a humanized anti-tau monoclonal antibody in clinical development for the treatment of PSP and AD. A phase I double-blind, placebo-controlled, single ascending dose trial (NCT02494024) to assess the safety, tolerability, and pharmacokinetics of ABBV-8E12 after intravenous infusion in 30 subjects affected by PSP has been recently completed [71]. Results showed a satisfactory safety and tolerability profile that supported further multiple-dose testing in patients with PSP, prompting a phase II clinical trial evaluating the efficacy and safety of ABBV-8E12 after 52 weeks in PSP subjects, which is currently recruiting (NCT02985879). The study intends to recruit 180 subjects who will meet eligibility criteria for possible or probable PSP, having symptoms for less than 5 years. Study completion date is expected to be during 2019. Besides this still-ongoing phase II study in PSP patients, an additional phase II2 clinical trial is also currently recruiting to evaluate ABBV-8E12 in early AD patients (NCT02880956). This repeated dose, multicenter, randomized, double-blind, placebo-controlled study to assess the ABBV-8E12 efficacy and safety in 400 subjects with early AD is expected to be completed by 2019–2020 [72]. At the beginning of 2018, an extension study of ABBV-8E12 in PSP to evaluate the long-term safety and efficacy of ABBV-8E12 in 340 subjects with PSP was registered (NCT03391765). Subjects that completed the 52-week treatment period in the phase II study mentioned above (NCT02985879) will be included.

The last passive immunotherapy approach to reach clinical development stage so far is R07105705 (RG6100), an anti-tau antibody for which very limited information is available regarding its precise nature or preclinical efficacy [73]. In 2016, a first phase I study of 71 volunteers comprising both healthy controls and people with mild to moderate AD began enrollment (NCT02820896). This single dose, dose-escalation, and multiple dosing study compares the antibody to placebo on safety, tolerability, pharmacokinetics, and preliminary activity outcomes. The study was expected to run until mid-2017. Although no results have been published, a 72-week phase II, randomized, placebo-controlled, double-blind study to assess the efficacy and safety of RO7105705 in 360 participants with prodromal to mild AD began recruiting in late 2017 (NCT03289143). Participants who complete the double-blind treatment period will be offered to enter into an optional 96-week open-label extension period.

In summary, quite a number of active and passive immunotherapies targeting tau protein are already in clinical trials (most likely with more to come) for treating not only AD, but also a primary tauopathy such as PSP (Table 1). As new tau-targeted immunotherapy strategies continue to be eagerly pursued, a number of key questions remain to be answered though, in particular with regards to the choice of immunogen, the tau species to be targeted, safety, and mechanism of action [14,58,74].

## 7. Future Outlook

As much as some open questions and challenges remain out in front, the data reviewed here are encouraging and demonstrate the potential of therapeutic efforts of tau-based strategies for the future treatment of tauopathies, including AD, either alone or in combination. Significant additional efforts are being made at the preclinical stage to validate a number of other tau-related targets different and complementary to those discussed in this review, with the aim of developing novel, perhaps better ways for the treatment of these conditions. However, the complexity of the tau protein misfolding, aggregation, and pathology propagation processes as well as the immune response during aging and neurodegeneration must be acknowledged so we can better design and develop safe and efficacious therapies. Lessons must be learned from the disappointing collective experience in the last 10–15 years to develop disease-modifying therapies for these disorders, so that we should proceed forward steadily but with caution in translating results from preclinical models into the clinical drug development stage. The existing growing interest in tau biology will definitely lead to a deeper understanding of tau’s biological functions and bring novel insights into the precise mechanisms and nature of the tau species responsible for neuronal dysfunction and its causative role in the neurodegenerative process. This will hopefully result in broadening the range of potentially useful therapeutic tools to treat such devastating conditions like AD and related tauopathies.

## Figures and Tables

**Table 1:** Compounds in clinical development for Alzheimer's disease (AD) and other tauopathies. GSK-3 = glycogen synthase kinase-3. PSP = Progressive Supranuclear Palsy. *O*-GlcNAc = *O*-Linked β-*N*-acetylglucosamine. PERK = double-stranded RNA-activated protein kinase-like endoplasmic reticulum kinase. MT = microtubule. CBD = Corticobasal degeneration. naPPA = non-fluent variant of primary progressive aphasia.

Compound	Mechanism of Action	Indication	Most Advanced Development Phase	Status	Trial ID	Reference
Tideglusib (NP031112, NP-12)	GSK-3 inhibitor	AD PSP	III	Completed	NCT00948259 NCT01350362 NCT01049399	[25,26,27]
Saracatinib (AZD0530)	Fyn inhibitor	AD	IIa	Ongoing	NCT01864655 NCT02167256	[30]
Salsalate	Acetylation inhibitor	PSP AD	Ib	Ongoing	NCT02422485 NCT03277573	[33]
MK-8719	*O*-GlcNAcase inhibitor	PSP	I	Ongoing	n.a.	[35,36]
ASN120290 (ASN-156)	*O*-GlcNAcase inhibitor	PSP	I	Ongoing	n.a.	n.a.
CCT020312	PERK Activator	PSP	I	Ongoing	n.a.	[38]
AZP2006	Stimulator of autophagy	PSP	I	Ongoing	n.a.	n.a.
Nilotinib	c-Abl inhibitor	AD	II	Ongoing	NCT02947893	[39]
Epothilone D (BMS-241027)	MT stabilizer	AD	I	Completed	NCT01492374	[40]
Abeotaxane (TPI-287)	MT stabilizer	CBD/PSP AD	I	Completed	NCT01966666 NCT02133846	n.a.
Davunetide (NAP, AL-108)	MT stabilizer	PSP naPPA	II/III	Completed	NCT01056965 NCT03174886)	[42,43]
Methylene Blue (Rember, TRx0014)	Aggregation inhibitor	AD	II	Completed	NCT00515333	[41]
LMTM (LMT-X, TRx0237)	Aggregation inhibitor	AD bvFTD	III	Ongoing	NCT01689246 NCT01626378	[51,52]
AADVac-1	Active immunotherapy	AD	II	Ongoing	NCT01850238 NCT02031198 NCT02579252	[56]
ACI-35	Active immunotherapy	AD	Ib	Ongoing	ISRCTN13033912	[57]
IVIg	Active immunotherapy	AD	III	Ongoing	NCT00818662 NCT01300728 NCT01561053	[58,59,60]
BMS-986168 (BIIB092, IPN007)	Passive immunotherapy	PSP	II	Ongoing	NCT02294851 NCT02460094 NCT02658916 NCT03068468	[62,63]
ABBV-8E12 (C2N-8E12)	Passive immunotherapy	ADPSP	II	Ongoing	NCT02494024 NCT02880956 NCT03391765 NCT02985879	[64,65]
R07105705 (RG6100)	Passive immunotherapy	AD	II	Ongoing	NCT02820896 NCT03289143	[66]
